# Prediction of Differentially Expressed Genes and a Diagnostic Signature of Preeclampsia via Integrated Bioinformatics Analysis

**DOI:** 10.1155/2022/5782637

**Published:** 2022-06-07

**Authors:** Shan Huang, Shuangming Cai, Huibin Li, Wenni Zhang, Huanshun Xiao, Danfeng Yu, Xuan Zhong, Pei Tao, Yiping Luo

**Affiliations:** ^1^Medical Intensive Care Unit, Guangdong Women and Children Hospital, Guangzhou, 510000 Guangdong, China; ^2^Pathology department, Guangdong Women and Children Hospital, Guangzhou, 510000 Guangdong, China

## Abstract

**Background:**

Preeclampsia (PE), which has a high incidence rate worldwide, is a potentially dangerous syndrome to pregnant women and newborns. However, the exact mechanism of its pathogenesis is still unclear. In this study, we used bioinformatics analysis to identify hub genes, establish a logistic model, and study immune cell infiltration to clarify the physiopathogenesis of PE.

**Methods:**

We downloaded the GSE75010 and GSE10588 datasets from the GEO database and performed weighted gene coexpression network analysis (WGCNA) as well as Gene Ontology (GO) and Kyoto Encyclopedia of Genes and Genomes (KEGG) analyses. The online search tool for the retrieval of interacting genes and Cytoscape software were used to identify hub genes, which were then used to establish a logistic model. We also analyzed immune cell infiltration. Finally, we verified the expression of the genes included in the predictive model via RT-PCR.

**Results:**

A total of 100 and 212 differently expressed genes were identified in the GSE75010 and GSE10588 datasets, respectively, and after overlapping with WGCNA results, 17 genes were identified. KEGG and GO analyses further indicated the involvement of these genes in bioprocesses, such as gonadotropin secretion, immune cell infiltration, and the SMAD and MAPK pathways. Additionally, protein-protein interaction network analysis identified 10 hub genes, six (*FLT1*, *FLNB*, *FSTL3*, *INHA*, *TREM1*, and *SLCO4A1*) of which were used to establish a logistic model for PE. RT-PCR analysis also confirmed that, except *FSTL3*, these genes were upregulated in PE. Our results also indicated that macrophages played the most important role in immune cell infiltration in PE.

**Conclusion:**

This study identified 10 hub genes in PE and used 6 of them to establish a logistic model and also analyzed immune cell infiltration. These findings may enhance the understanding of PE and enable the identification of potential therapeutic targets for PE.

## 1. Introduction

Preeclampsia (PE), which is characterized by proteinuria and hypertension, is a potentially dangerous syndrome that occurs in pregnant women after 20 weeks of gestation [1]. It may cause several complications, including premature birth, abortion, HELLP syndrome, renal function damage, and eclampsia [2]. Additionally, with a high incidence rate worldwide (3–5%), it represents a significant danger to the health of pregnant women and newborns and is one of the leading causes of maternal and neonatal deaths [3, 4]. Therefore, understanding its pathogenesis, developing methods for its early diagnosis, and studying effective treatment measures for its management are important for protecting the lives of pregnant women and perinatal children and also conserving public resources.

The placenta plays an important role in the pathogenesis of PE [5]. Specifically, the shallow invasion of the placenta is an important factor in PE, as it causes long-term ischemia and hypoxia in trophoblasts. This process continues until the second trimester. Without adequate blood supply, the trophoblasts release inflammatory factors into maternal blood, leading to an increase in maternal blood pressure and causing damage to certain organs [6]. Presently, several theories, such as angiogenesis disorder [7], immune dysfunction [8], inflammasome activity [9], and senescence [10], have been proposed in an attempt to explain this phenomenon. However, the exact cause of shallow placental invasion is still unclear [11].

In recent years, bioinformatics analysis and the microarray technology have been used to identify transcriptomic alterations and differentially expressed genes (DEGs), as well as their physiological functions, in many diseases. This has led to enhanced understanding regarding the pathophysiological processes of these diseases [12, 13]. Additionally, in several previous studies, PE was investigated using bioinformatics analyses. Specifically, Wu et al. observed that *RAD21*, *UBC*, *SUMO1*, and *SUMO2* may be reliable biomarkers of PE [14], while the results of a study conducted by Kang et al. indicated that *CCR7* and *ITGA5* may play important roles in the early onset of PE [15]. However, studies with a focus on the establishment of a logistic regression model and assessment of immune cell infiltration in PE are limited.

Thus, in this study, we used the Gene Expression Omnibus (GEO) datasets GSE75010 [16] and GSE10588 [17] to identify hub genes and establish a logistic regression model for the early diagnosis of PE. Further, we also used RT-PCR to test the mRNA expression levels of the identified genes and also studied immune cell infiltration in PE using the abovementioned datasets.

## 2. Materials and Methods

### 2.1. Microarray Data Collection and Preprocessing

The placental mRNA profiles in the GSE75010 and GSE10588 datasets were downloaded from the GEO database (https://www.ncbi.nlm.nih.gov/geo/). Specifically, from the GSE75010 dataset, transcriptional profiles corresponding to 80 PE placentas and 77 non-PE placentas were generated using the GPL6244 platform of the Affymetrix Human Gene 1.0 ST Array, and from the GSE10588 dataset, transcriptional profiles corresponding to 17 PE placentas and 26 non-PE placentas were generated using the GPL2986 platform of the ABI Human Genome Survey Microarray Version 2. Further, R software was used to transform the probe numbers to gene symbols and remove the null probes.

### 2.2. Identification of DEGs

To identify DEGs between placenta tissue samples from normal pregnant women and women with PE in both datasets, we used the “limma” R software package. Thereafter, the DEGs were visualized using heatmaps and volcano plots.

### 2.3. Weighted Gene Coexpression Network Analysis (WGCNA)

A weighted coexpression network was constructed using the “WGCNA” R software package. Specifically, the minimal module size was set to 50, and the cut height was set to 0.25. For both the GSE75010 and GSE10588 datasets, the soft-thresholding power was set to 2.

### 2.4. Identification of Overlapping Genes and Functional Classification of These Genes

The DEGs and genes in the most relevant WGCNA modules based on both datasets were compared using the R software package “VennDiagram.” Seventeen overlapping genes were identified. Kyoto Encyclopedia of Genes and Genomes (KEGG) pathway and Gene Ontology (GO) term analyses were performed using the R software package “ClusterProfiler” to evaluate the function of the overlapping genes. Here, statistical significance was set at *P* < 0.05.

### 2.5. Protein-Protein Interaction (PPI) Network Analysis

For PPI network analysis, we used the search tool for the retrieval of interacting genes (STRING) (http://string-db.org), with the cutoff criterion set at 0.4. We also used Cytoscape software to visualize the PPI network, and finally, to identify hub genes, we used CytoHubba.

### 2.6. Construction of a Logistic Regression Model

The samples corresponding to the GSE75010 dataset were randomly divided into two groups: the training group (60%) and the test group (40%). The clinical features of these two groups were the same (*P* > 0.05). The logistic regression model was established using the “glmnet” package in R, based on the training group data and was validated using the test group data. Further, the receiver operating characteristic (ROC) curve was drawn using the pROC package in R, while the area under the ROC curve (AUC) was determined using the auc () function in R language. Further, principal component analysis (PCA) was performed using the prcomp () function to test whether the DEGs or hub genes in the logistic model could distinguish normal pregnant women from patients with PE.

### 2.7. Immune Infiltration Analysis and Immune Scores

The R language source code for immune infiltration analysis was downloaded from CIBERSORT and used to assess the relative proportions of 22 immune cells in each sample from the GSE75010 dataset. Samples with *P* < 0.05 were selected. Thereafter, the immune infiltration results were visualized using R language in the form of heatmaps, bar plots, and coheatmaps.

### 2.8. Quantitative Real-Time RT-PCR

Placental tissues from three patients with PE and three women with normal pregnancy at the same gestational week were collected at our hospital during delivery. All of the participants provided written informed consent for their tissue samples to be used in this study. Further, the study was approved by the Ethics Committee of Guangdong Women and Children Hospital and was performed in accordance with the principles outlined in the Declaration of Helsinki.

After the collection of the placental tissue samples, TRIzol reagent (Invitrogen, Carlsbad, CA, USA) was used to isolate the placental tissue total RNA according to the manufacturer's instructions. Reverse transcription was then performed using the Revert Aid RT-PCR system, and real-time PCR was performed using the ABI 7500 Real-Time PCR System (Roche, Penzberg, Germany) by mixing primers, cDNA, and the Rox Reference Dye. The conditions for the RT-PCR were as follows: 40 cycles of denaturation (95°C, 10 s), annealing (55°C, 20 s), and extension (72°C, 35 s). The primer sequences were as follows: *FLT1* (forward, 5′-CCGGCTCTCTATGAAAGTGAAG-3′; reverse, 5′-CGAGTAGCCACGAGTCAAATAG -3′), *FLNB* (forward, 5′-CCCTCGCTCTGGTGATTATTT-3′; reverse, 5′-AAGGGACTGAAACGGACTTG-3′), *FSTL3* (forward, 5′-TTGATGCTCAGAATCGCCTAC-3′; reverse, 5′-TATCCTCCGTGTTGTCCTCT-3′), *INHA* (forward, 5′-CTCGGATGGAGGTTACTCTTTC-3′; reverse, 5′-CACCAGCCATGGGATTAAGA-3′), *TREM1* (forward, 5′-CCCAGCATTGTTCCTGTTTATG-3′; reverse, 5′-TCTGCCTCTCCTAGAGTGTATT-3′), *SLCO4A1* (forward, 5′-GGTGGGAGGAACTTGCATAA-3′; reverse, 5′-CCACACACGATCGGGTATAAA-3′), and *GAPDH* (forward, 5′-CAAGAGCACAAGAGGAAGAGAG-3′; reverse, 5′-CTACATGGCAACTGTGAGGAG-3′). The mRNA expression levels of the hub genes were calculated using the *ΔΔ*CT method with GAPDH as a reference.

### 2.9. Statistical Analysis

All statistical analyses were performed using R software version 4.0.0, and *P* < 0.05 was considered statistically significant.

## 3. Results

### 3.1. Identification of DEGs in PE

**Figure 1:**
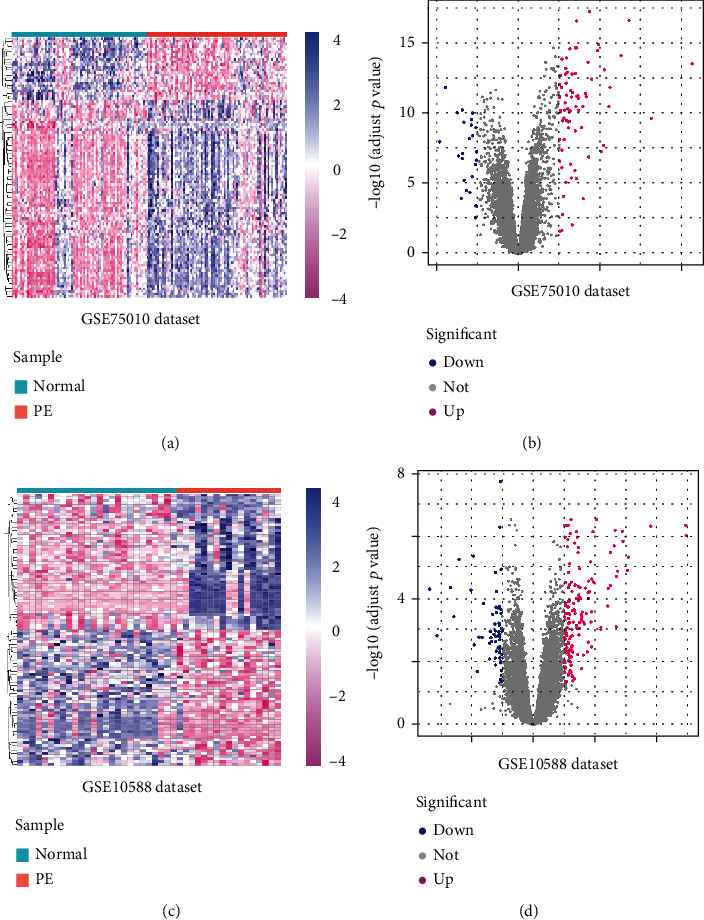
Results of the analysis of differentially expressed genes (DEGs) between placental tissues from normal pregnant women and patients with preeclampsia based on the GSE75010 and GSE10588 datasets. (a) Heatmap and (b) volcano plot of DEGs in the GSE75010 dataset. (c) Heatmap and (d) volcano plot of DEGs in the GSE10588 dataset.

To identify the DEGs between normal pregnant women and women with PE, we used the GSE75010 dataset, which comprises transcriptional profiles from 77 control pregnant women and 80 patients with PE. Using the “limma” R software package, 100 DEGs, including 76 upregulated and 24 downregulated genes, were identified (Figures 1(a) and 1(b)). Similarly, we analyzed the GSE10588 dataset, which comprises transcriptional profiles corresponding to 26 control pregnant women and 17 patients with PE, and identified 212 DEGs, including 153 and 59 upregulated and downregulated genes (Figures 1(c) and 1(d)). The first 50 DEGs in both datasets are displayed via volcano plots and heatmaps.

### 3.2. Weighted Gene Coexpression Network Analysis (WGCNA)

**Figure 2:**
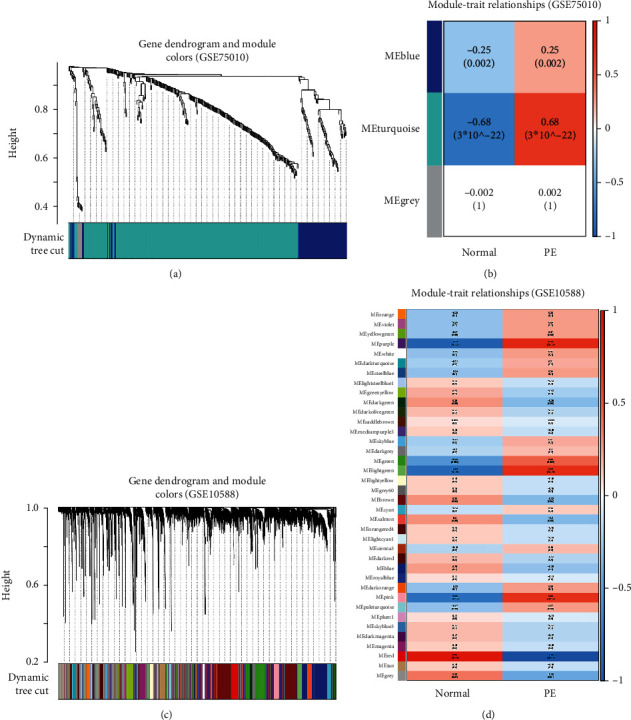
WGCNA results of the GSE75010 and GSE10588 datasets. (a) Gene dendrogram and module colors based on the GSE75010 dataset. (b) Module-trait relationships based on the GSE75010 dataset. (c) Gene dendrogram and module colors based on the GSE10588 dataset. (d) Module-trait relationships based on the GSE10588 dataset.

After identifying the DEGs, we used WGCNA to determine the most relevant modules with respect to PE in the GSE75010 and GSE10588 datasets. Specifically, in the GSE75010 dataset, we set the soft-thresholding power to 2 to establish a scale-free gene coexpression network and generated three modules using the dynamic tree-cut algorithm. We observed that the turquoise module, which includes 295 genes, was the most negatively regulated module in the normal group; thus, it was selected for subsequent analysis (Figures 2(a) and 2(b)). Further, in the GSE10588 dataset, setting the soft-thresholding power to 2 generated 38 modules, with the purple module, including 354 genes, being the most negatively regulated module in the normal group. Thus, it was also selected for further analysis (Figures 2(c) and 2(d)).

### 3.3. Overlapping Genes in the Two Datasets

**Figure 3:**
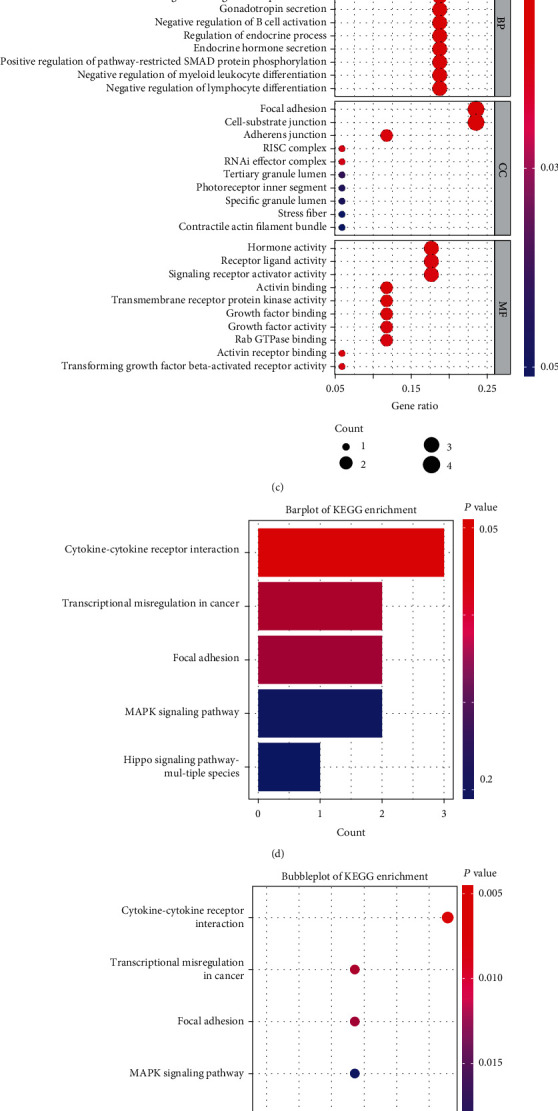
Identification of hub genes and the results of GO, KEGG, and PPI network analyses. (a) Venn diagram showing DEGs and WGCNA modules based on the GSE75010 and GSE10588 datasets. (b and c) Bar plot and bubble plot showing overlapping genes based on GO analysis. (d and e) Bar plot and bubble plot of overlapping genes based on KEGG analysis. (f) PPI network of the overlapping genes. (g) The most significant top 10 hub genes in the PPI network.

To further identify PE-related genes, we used the “VennDiagram” package in R software to draw a Venn diagram showing the DEGs and the genes in the most negative and relevant modules corresponding to the control groups from the GSE75010 and GSE10588 datasets. Seventeen overlapping genes were identified as most relevant to PE (Figure 3(a)).

### 3.4. GO and KEGG Analyses of Overlapping Genes

To better understand the function of the overlapping genes, we subjected the 17 overlapping genes to KEGG and GO analyses. The five most significantly enriched biological process terms were “regulation of gonadotropin secretion,” “gonadotropin secretion,” “negative regulation of leukocyte differentiation,” “negative regulation of hematopoiesis,” and “negative regulation of B-cell activation.” Further, the five most significantly enriched molecular function terms were “activin binding,” “hormone activity,” “transmembrane receptor protein kinase activity,” “growth factor binding,” and “receptor-ligand activity.” Furthermore, the five most significantly enriched cellular component terms were “focal adhesion,” “cell-substrate junction,” “adherens junction,” “RISC,” and “RNAi effector complex” (Figures 3(b) and 3(c)).

Additionally, using KEGG pathway enrichment analysis, we observed that five pathways were enriched, namely, “cytokine-cytokine receptor interaction,” “transcriptional misregulation in cancer,” “focal adhesion,” “MAPK signaling pathway,” and “Hippo signaling pathway” in multiple species (Figures 3(d) and 3(e)).

### 3.5. PPI Network Analysis and Identification of Hub Genes

From the DEGs and WGCNA modules obtained in the previous steps, we identified 17 overlapping genes based on the two datasets employed in this study as most relevant to PE. Next, to identify the hub genes of these 17 overlapping genes, we uploaded them to the STRING online database and used the Cytoscape software to generate a PPI network, which included 16 nodes (Figure 3(f)). Next, the use of CytoHubba to identify hub genes revealed that the top 10 hub genes were *INHA*, *ENG*, *INHBA*, *FLT1*, *FLNB*, *FSTL3*, *LEP*, *NDRG1*, *ISL1*, and *TREM1* (Figure 3(g)).

### 3.6. Logistic Regression Model

**Figure 4:**
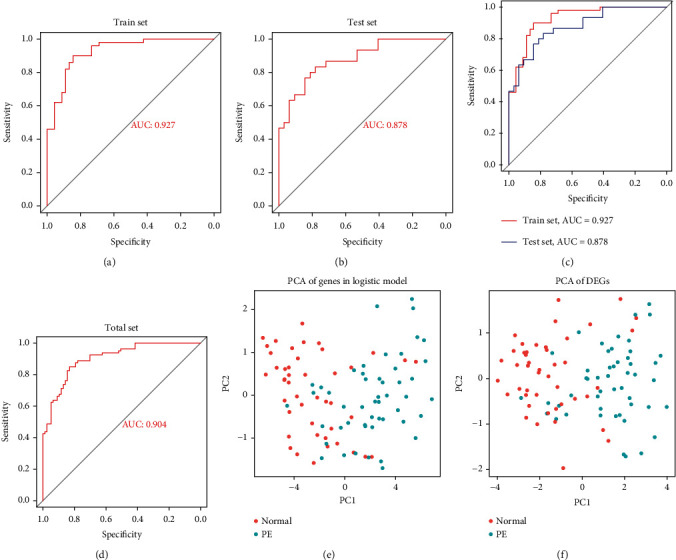
The logistic regression model. (a–c) AUCs corresponding to the training group and test group. (d) AUC based on the GSE75010 dataset. (e and f) PCA of the DEGs and the genes in the logistic model.

We next constructed a logistic regression model. Specifically, we randomly separated the GSE75010 dataset into two groups, namely, the training group and test group, with matched clinical features (*P* > 0.05). Thereafter, we used the training group to perform a logistic regression analysis and observed that the *P* values corresponding to *FLT1*, *FLNB*, *FSTL3*, *INHA*, *TREM1*, and *SLCO4A1* were below 0.05. Further, the AUC corresponding to the training group was 0.927, while that corresponding to the test group was 0.878 (Figures 4(a)–4(d)). We also observed that via PCA of the DEGs and the genes in the logistic regression model, it was possible to distinguish placental tissues from normal pregnant women from those corresponding to their counterparts with PE.

### 3.7. Immune Cell Infiltration

**Figure 5:**
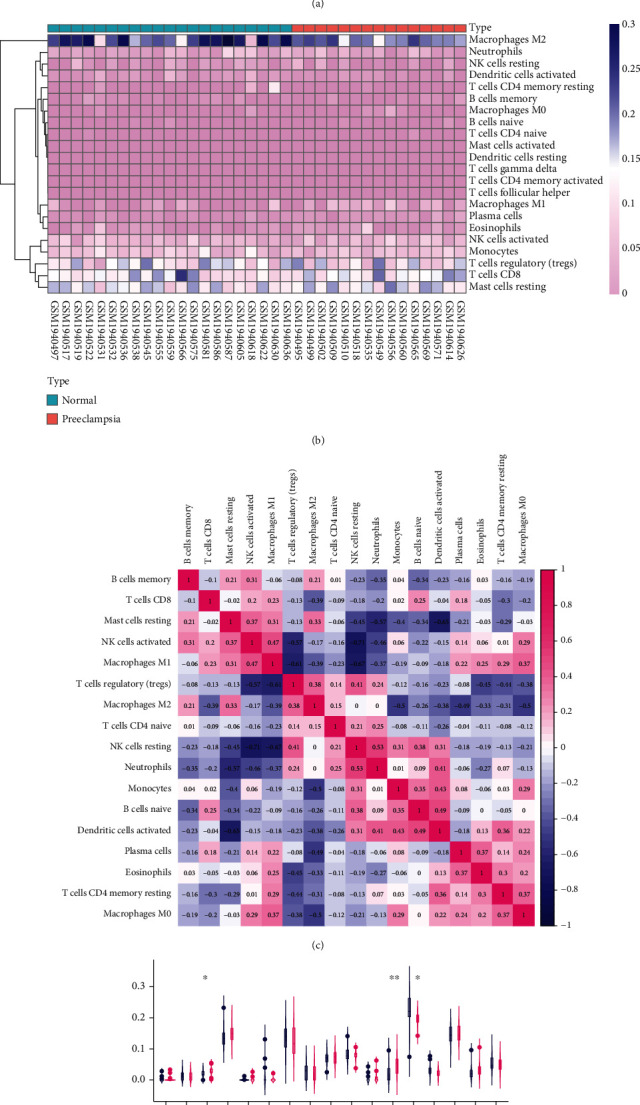
Immune cell infiltration analysis. (a) Relative percentage of immune cells in samples from the GSE75010 dataset. (b) Heatmap of immune cells. (c) Correlation analysis of immune cells. (d) Comparison of immune cell infiltration between normal pregnant women and patients with PE.

PE is closely associated with immune response. To examine the changes in immune cell infiltration in the placenta in pregnant women with PE, we analyzed the GSE75010 dataset for differences in infiltration in terms of 22 immune cell types using the CIBERSORT method. To this end, *P* < 0.05 was considered to be statistically significant for each sample, and data corresponding to 21 normal pregnant and 15 patients with PE were included in analysis. We observed that three immune cell types displayed the highest differential infiltration between normal pregnant women and pregnant women with PE. Specifically, in patients with PE, plasma cells and M1 macrophages were upregulated, while M2 macrophages were downregulated (*P* < 0.05) (Figure 5).

### 3.8. RT-PCR

**Figure 6:**
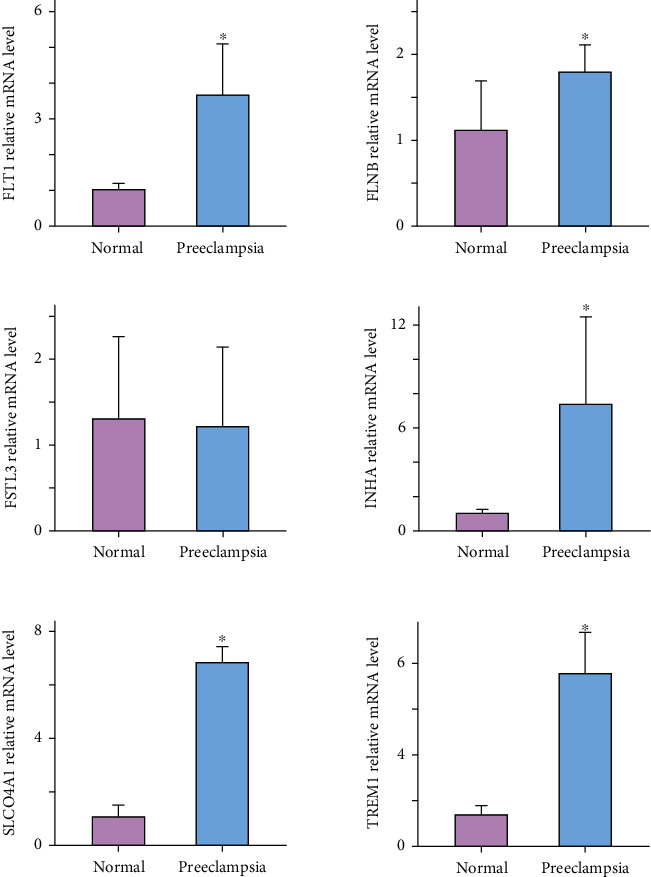
Relative expression levels of the genes identified via the logistic regression analysis involving normal placental tissues and placental tissue from patients with PE. ^∗^*P* < 0.05 compared with the normal pregnant women group. The error bars represent the standard deviation of the measurements based on triplicate runs.

We collected placental tissues from normal pregnant women and women with PE, and via RT-PCR, we investigated the relative gene expression levels of the six genes in the logistic model. We observed that *FLT1*, *FLNB*, *INHA*, *TREM1*, and *SLCO4A1* showed increased expression levels in the PE group relative to their expression levels in the normal group (Figure 6).

## 4. Discussion

The aim of this study was to identify hub genes in PE to the end of clarifying the mechanism of its pathogenesis and developing a logistic model. We also studied immune cell infiltration in patients with PE.

PE, which is characterized by hypertension and positive urinary protein in women after 20 weeks of pregnancy, can lead to organ function damage and tends to worsen with an increase in gestational weeks. Thus, it is a serious threat to the health of mothers and newborns. Currently, because its pathogenesis is unclear, the only effective treatment for PE is pregnancy termination [18, 19]. Thus, conducting studies to provide clarification in this regard and developing effective treatments for its management are of prime importance. The development of microarray analysis and the RNA-seq technology has enabled measurement of the expression of many genes as well as the identification of DEGs between normal and patient tissues. This is important for understanding disease mechanisms [20].

WGCNA is an analytical method used to analyze the gene expression patterns of multiple samples. It can cluster genes with similar expression patterns, analyze the relationship between modules and specific traits or phenotypes, and finally identify target genes and gene networks for disease treatment [21]. Compared with traditional analytical methods, this new processing method has the potential for application in bringing correlation values more in line with the characteristics of scale-free networks, thereby providing more biologically significant results [22]. In this study, we used WGCNA to identify the most negative modules corresponding to the control group in each dataset and identified 17 overlapping genes that were the most differentially expressed.

To further investigate the function of the overlapping genes, we performed KEGG and GO analyses and observed the enrichment of some important biological processes, including gonadotropin secretion. Several studies have been conducted to investigate the effect of gonadotropin in PE. For example, Li et al. observed that the expression of the mRNA of the FSH receptor is reduced in PE [23], while Reisinger et al. reported that gonadotropins, such as FSH and LH, are angiogenic factors and play an important role in PE [24]. Another biological process associated with PE is the SMAD pathway, which is well known for its relationship with epithelial mesenchymal transition (EMT) and angiogenesis. Several studies have demonstrated that Smad2, Smad4, and Smad7 may participate in PE via the EMT pathway [25, 26]. In this study, we also observed that several biological processes that showed enrichment based on the GO analysis, including “negative regulation of B-cell activation” and “negative regulation of lymphocyte differentiation,” were associated with immune cell infiltration. Therefore, we studied the relationship between immune cells and the DEGs to clarify the existence of an association between PE and immune cell infiltration. Our analyses suggested that M1 macrophages were upregulated in patients with PE. Additionally, it has been reported that macrophages play an essential role in regulating immune response, which is important in the pathogenesis of PE [27]. Several studies have also been conducted to clarify the function of these immune cells in PE, and it has been suggested that macrophages mediate the apoptosis of extravillous trophoblasts and also maintain maternal-fetal tolerance [28]. Further, it has also been hypothesized that changes in macrophage dysfunction and polarity may induce PE.

After identifying hub genes, we screened six genes, namely, *FLT1*, *FLNB*, *FSTL3*, *INHA*, *TREM1*, and *SLCO4A1*, to establish the logistic model. This model represented a screening technique that could be used to distinguish patients with PE from women with normal pregnancies. Further, Fms-related tyrosine kinase 1, also known as FLT1 or VEGFR1, is encoded by *FLT1* in the human body, and Flt1, which is a member of the src gene family, is related to the oncogene, reactive oxygen species [29], and exhibits tyrosine protein kinase activity, which is involved in the control of cell differentiation and proliferation. Furthermore, sFLT1, a soluble Flt1 protein, is an antiangiogenic factor originating from the placenta [30], and its overproduction is an important event that drives the clinical features of PE, such as hypertension. Several scholars have shown that the mRNA level of sFLT1 is upregulated in both the blood and placenta of patients with PE [31], suggesting that sFLT1 is a potential predictive factor for PE [32].

Recently, it was observed that FLNB, which is thought to be a dimeric actin-binding protein that is implicated in skeletal deformities, plays a role in platelet dysfunction and hypertension; however, very little is known regarding its role in PE. In this study, *FLNB* was identified as a hub gene for PE; hence, its effect on PE requires further research [33, 34].

*INH*, which is also known as inhibin, is a glycoprotein hormone that comprises two subunits, the *α* and *β* subunits. Specifically, the *α* subunit, which is expressed in a variety of human tissues, such as the placenta, determines its specificity. Depoix et al. observed that *INHA* is associated with PE [35]. Additionally, TREM1, which is a myeloid cell surface receptor that is expressed on the surfaces of neutrophils, monocytes, and macrophages, amplifies inflammatory responses in coordination with classical pattern recognition receptors (PRRS), such as toll-like receptor (TLR) family and nod-like receptor (NLR) family. Xie et al. reported that during PE, TREM1 amplifies trophoblastic inflammation via the activation of the NF-*κ*B pathway [36]. Additionally, studies on *SLCO4A1* have been predominantly focused on microRNA and cancer. There are no reports in this regard on PE; thus, further studies are needed to clarify its effect on PE.

In conclusion, in this study, using the WGCNA method, we identified 10 hub genes associated with PE, and after GO, KEGG, PPI network, and immune infiltration analyses involving these genes, six (*FLT1*, *FLNB*, *FSTL3*, *INHA*, *TREM1*, and *SLCO4A1*) were selected to construct a logistic model. We observed that overexpression of *FLT1*, which is an antiangiogenic factor originating from the placenta, is an important event that drives the clinical feature of PE. It was also identified as a potential predictor for PE. Further, *INHA* and *TREM1* were also found to be associated with PE. Therefore, further studies on these three classic PE-related genes may reveal the pathogenesis of PE, facilitate the identification of potential therapeutic targets and strategies for early diagnosis, and also accelerate the development of new effective therapies. Interestingly, studies on the roles of *FLNB* and *SLCO4A1* in PE are limited. RT-PCR showed that these two genes were upregulated in patients with PE. Therefore, in future, further studies should focused on clarifying their functional and diagnostic values in PE. This will provide new ideas regarding the mechanism of the pathogenesis of PE.

## 5. Conclusions

Taken together, the outcomes of this study enhance the understanding regarding the pathophysiological mechanisms of PE and also clarify the identification of potential therapeutic targets for PE and the development of diagnostic methods for its early diagnosis. In future, it would be necessary to focus on the functional and diagnostic values of *FLNB* and *SLCO4A1* in PE.

## Data Availability

The GSE75010 and GSE10588 placental mRNA profiles were downloaded from the GEO database (https://www.ncbi.nlm.nih.gov/geo/).
